# The NuA4/TIP60 histone-modifying complex and Hr78 modulate the *Lobe*^*2*^ mutant eye phenotype

**DOI:** 10.1515/biol-2025-1164

**Published:** 2025-08-20

**Authors:** Madison P. Stonbraker, Dominic A. DePaul, Ethan C. Heidel-Roberts, Hallee Greene, Matthew Logan Johnson

**Affiliations:** Biology Department, University of Pittsburg at Greensburg, Greensburg, Pennsylvania, United States of America

**Keywords:** GAL4/UAS, Jazf-1, Hr78, enhancer, suppressor

## Abstract

Gene regulation is important during tissue formation, but redundant systems make it difficult to study *in vivo.* The protein Jazf-1 is a member of the NuA4/TIP60 histone-modifying complex, and a transcriptional repressor has been suggested to be important for *Drosophila melanogaster* eye development. We used the GAL4-UAS system to determine the impact of altering gene expression. GAL4-UAS manipulations of Jazf-1 in the eye caused variable and not fully penetrant phenotypes. Increased expression of Jazf-1 has been shown to suppress a *Lobe* ^*2*^ small eye phenotype. We found that *Lobe* ^*2*^ produces a sensitive background for an *in vivo* assay to monitor gene regulatory complexes. Depleting Jazf-1 and other NuA4/TIP60 complex members significantly enhanced the eye phenotype. We also tested Hr78, which directly interacts with Jazf-1, and found it inversely modifies the *Lobe* ^*2*^ phenotype. An Hr78 mutation predicted to uncouple the Jazf-1 interaction but still capable of interactions with transcriptional activators further enhanced the *Lobe* ^*2*^ mutant phenotype, suggesting the loss of a repressing complex. We believe that Hr78 is acting as an anchor for repressing and activating complexes and the NuA4/TIP60 complex helps repress genes that can negatively impact eye formation in the context of *Lobe* ^*2*^.

## 1 Introduction

Jazf-1 is a transcription factor that has been implicated in both disease pathogenesis and core cellular processes. Certain alleles in humans are widely associated with diabetes susceptibility [1,2,3], and *de novo* translocations of Jazf-1 are the largest cause of Endometrial Stromal Sarcomas [4,5]. Beyond health implications, the dependance of core cellular processes, such as ribosome biogenesis, on Jazf-1 has been demonstrated in human systems [6]. Furthermore, Jazf-1 is predicted to be an early regulator in the formation of different tissues, such as the nervous system and eyes, in *Drosophila melanogaster* [7]. In support of the role for Jazf-1 in eye formation, it has previously been detected in a screen for genes that could suppress a mutation in the gene *Lobe* [8].

Lobe (*L*) is a transcription factor (Lobe/Oaz) with a classic dominant mutation, *L* ^*2*^, that causes smaller eyes [9]. The *L* ^*2*^ allele was recently determined to be caused by the presence of a transposable element [10]. This hypermorphic allele results in a smaller eye phenotype due to the overexpression of the *Lobe* gene [10]. Previous genetic screens with *L* ^*2*^ have investigated enhancers and suppressors to the *L* ^*2*^ allele when gene expression was controlled under an *eyeless* driver [8]. One of the 33 genes that were found to act as a suppressor of the *L* ^*2*^ mutant phenotype was the gene Jazf-1 (CG12054) [8]. Surprisingly, even though thousands of lines were tested, no other proteins that are known to physically associate with Jazf-1 were detected in this screen [6,11,12,13].

Jazf-1 is part of the NuA4/TIP60 histone-modifying complex in both mammalian and *D. melanogaster* cells [6,11,12]. Recent studies have demonstrated that in both humans and *D. melanogaster*, NuA4/TIP60 is responsible for acetylation of Lysine 12 on Histone H4 (H4K12ac), through the histone acetyl transferase TIP-60 [6,12]. RNAi knockdowns of Jazf-1 have also been demonstrated to negatively impact the level of H4K12ac [6]. The NuA4/TIP60 complex also has approximately 14 identified members, including unique members such as TRRAP/Nipped-A and Xbp-1 [12,14,15]. Loss of TRRAP/Nipped-A has been shown to negatively impact the ability of the complex to access different areas of the genome [16]. Jazf-1 has repeatedly been shown to be part of a stable subunit within the NuA4/TIP60 complex [17]. Finally, several members from both complexes were recently identified as being important during tissue formation, including the eye, further supporting the idea that Jazf-1 could regulate eye formation, as part of the NuA4/TIP60 complex [18].

In addition to its role in the NuA4/TIP60 complex, Jazf-1 has been found with NR2C2 (TAK1/TR4/TIP27), a nuclear receptor protein [19]. The interaction between Jazf-1 and NR2C2 was initially detected over 20 years ago via yeast two-hybrid [19]. Since then, it has been repeatedly shown to be an important interacting partner of Jazf-1, and most recently, the interaction has been extensively characterized using X-ray crystallography [19,20,21,22]. Furthermore, Jazf-1 seems to act primarily as a repressor; however, there is conflicting evidence that Jazf-1 may act as a repressor or a co-activator with NR2C2 [6,17]. This interaction occurs through binding with the activation function 2 (AF-2) domain on the C-terminus, a region commonly used by transcription activators [23,24]. While most activators interact through the AF-2 domain of NR2C2, some, such as p300/CREB binding protein, interact through the activation function 1 (AF-1) domain located on the N-terminus of the protein [25]. The nuclear receptors are highly conserved, and Hr78 in *D. melanogaster* is its closest homolog [26,27]. Like NR2C2, Hr78 was also detected in a yeast two-hybrid screen as having a direct interaction with Jazf-1 in *D. melanogaster* [13].

While Jazf-1 has been extensively studied biochemically, it is not as well defined genetically [6,12,17,21,28]. To this end, the GAL4-UAS binary expression system has two important features to allow tissue-specific manipulation: the driver and the responder. The driver is responsible for the special and temporal expression of the GAL4 protein, a transcription factor. The driver is often coupled to a specific gene promoter to mimic the endogenous expression of that gene. The responder is the gene that will be activated, as it is located immediately downstream of a DNA sequence known as an upstream activating sequence (UAS). The UAS sequence is a specific DNA sequence that the GAL4 protein binds, leading to transcriptional activation [29].

Given the growing evidence for the role of Jazf-1 in the eye, we wanted to investigate the contribution of Jazf-1 and other members of the NuA4/TIP60 complex during eye development. To do this, we genetically manipulated the expression of Jazf-1 within the *D. melanogaster* eye. Additionally, we tested NuA4/TIP60 complex members in the context of a sensitive *L* ^*2*^ mutant background. Furthermore, we tested manipulations of Hr78 to determine if this would also have any developmental consequences for the *L* ^*2*^ mutant phenotype.

## 2 Methods

### 2.1 Stocks used

All stocks used were obtained through the Bloomington Drosophila Stock Center. GFP expression in the Jazf-1 locus (RRID:BDSC_605433) (y^1^ w^*^; TI[GFP^[3xP3.cLa]^ = CRIMIC.TG4.1]CG12054^[CR71307-TG4.1]^/TM3, Sb^1^ Ser^1^) [30]. *Lobe* ^*2*^ mutant lines are a spontaneous mutant (RRID: BDSC_319) (*L* ^*2*^/CyO) [9,10]. *Hr78* ^*2*^ mutant lines contain an early stop codon in *Hr78* (RRID: BDSC_4436) (w^*^; Hr78^2^/TM6B, Tb^+^) [31]. The *eyeless* GAL4 drivers (RRID:BDSC_8220) (y^1^ w^1118^; P[w^[+mC]^ = ey3.5-GAL4.Exel]2) and (RRID:BDSC_8227) (y^1^ w^1118^; P[w^[+mC]^ = ey3.5-GAL4.Exel]3) were used as either second or third chromosome drivers respectfully, and we referred to them as *eyGAL4*. Stocks for overexpression of Jazf-1 (UAS:Jazf-1) (RRID:BDSC_15535) (y^1^ w^67c23^; P[EPgy2]CG12054^EY01782^) [32] and Hr78 (UAS:Hr78) (RRID: BDSC_27214 (y^1^ w^*^; P[EP]Hr78^G6746^) were also used [33]. The RNAi lines used for knockdown were Jazf-RNAi (RRID: BDSC_50910) (y^1^ v^1^; P[TRIP.HMJ03134]attP40), Xbp-1-RNAi (RRID: BDSC_36755) (y^1^ sc^*^ v^1^ sev^21^; P[TRiP.HMS03015]attP2), Nipped-A-RNAi (BDSC: 34849) (y^1^ sc^*^ v^1^ sev^21^; P[TRiP.HMS00167]attP2), and Hr78-RNAi (RRID: BDSC_31990) (y^1^ v^1^; P[y^[+t7.7]^v^[+t1.8]^ = TRiP.JF03424]attP2 [34].

### 2.2 Genetic crosses

UAS-GAL4-manipulated *D. melanogaster* was crossed as follows. Virgin female L^2^/CyO; eyGAL4/eyGAL4 flies were crossed with males containing a UAS responder line (either UAS overexpressing or UAS-RNAi lines). Female offspring were measured as eyGAL4 heterozygotes with the appropriate UAS responder, and either containing the *L* ^*2*^ or CyO chromosome.

### 2.3 Growth conditions

All flies were raised in vials with standard R media [35] purchased from Lab Express (Ann Arbor, MI). Adults were limited to no more than 20 females and no more than 10 males per bottle, and progeny were raised at 25°C with 40–60% humidity.


**Ethical approval:** The research related to animal use has been complied with all the relevant national regulations and institutional policies for the care and use of animals.

### 2.4 Genetic crosses

All flies were manipulated under standard CO_2_ conditions using genetic markers from standard balancer chromosomes [36].

### 2.5 Fluorescent and brightfield microscopy

Zeiss Primo Star trinocular microscopes, Moticam Pro 205A, and Motic Images Plus 3.0 software were used to analyze *D. melanogaster* lines. Fluorescent microscopy was performed using freshly frozen GFP-expressing flies or those with a UAS:GFP responder and no driver. Flies containing GFP genes were exposed to florescence under a 480 nm filter cube for 30 ms. Brightfield microscopy was used to measure the length of *D. melanogaster* eyes along the dorsal–ventral axis. In general, flies were frozen prior to use on the microscope and one eye from each fly was measured with the genotype blinded to the researcher making the measurements. Female flies were used for all the data reported due to greater variability with *L* ^*2*^ in male flies.

### 2.6 Statistics

Statistical analysis was performed using a student *t*-test and ANOVA (Excel Software). *P*-values less than 0.05 were considered significant. All measurements were based on 10 female adults, and standard deviation was used for error bars on graphs.

## 3 Results

### 3.1 Jazf-1 is expressed in the adult eye and ocelli photoreceptors

**Figure 1:**
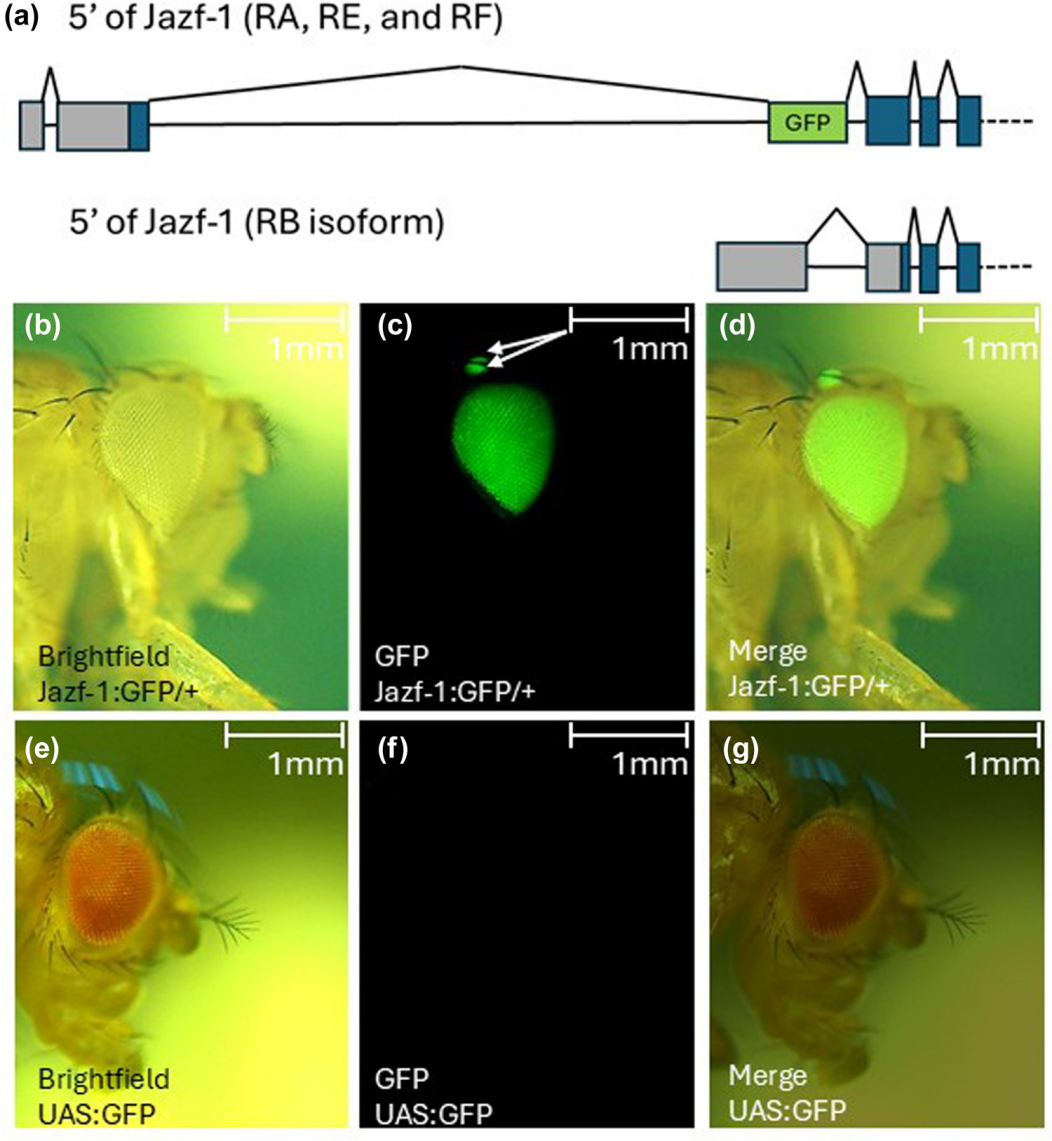
Jazf-1 locus with GFP expression. (a) Schematic representation of GFP inserted into the Jazf-1 locus. RA, RE, and RF coding sequences will splice in the GFP cassette. Jazf-1 RB is incapable of splicing in the GFP cassette. (b–g) Jazf-1 expression in *Drosophila melanogaster* eyes and ocelli photoreceptors. (b–d) UAS:GFP responder and a Jazf-1:GAL4 driver were inserted in the Jazf-1 locus. (e–g) UAS:GFP responder inserted in a different location with not responder. Brightfield (b and e), GFP (c and f), and merge (d and g) images demonstrate that expression from the Jazf-1 locus but not responder controls in the eye and ocelli (white arrows).

To investigate the expression of Jazf-1 in the eye, we used a publicly available inserted GFP trap into the Jazf-1 locus. Briefly, the TI[CRIMIC.TG4.1] is inserted by CRISPR/Cas9 followed by recombination-mediated cassette exchange to insert a GFP trap with a GAL4 trap [37]. This recombination-mediated exchange introduces a splice site to the GFP cassette following the first coding exon of three of the four annotated Jazf-1 isoforms to express GFP (Jazf-1-RA, RE, and RF but not RB) (Figure 1a). This allows expression levels of GFP to be consistent with endogenous expression of Jazf-1 RNA. The Jazf-1 gene trap line coupled with a GFP marker allowed us to investigate gene expression of Jazf-1 within the accumulation of GFP-marked cells. GFP expression using this system was robustly detected in the eyes of *Drosophila.* In addition, to the eyes, the ocelli, a set of photoreceptors on the top of the *Drosophila* head, robustly expressed GFP under the Jazf-1 locus (Figure 1).

**Figure 2:**
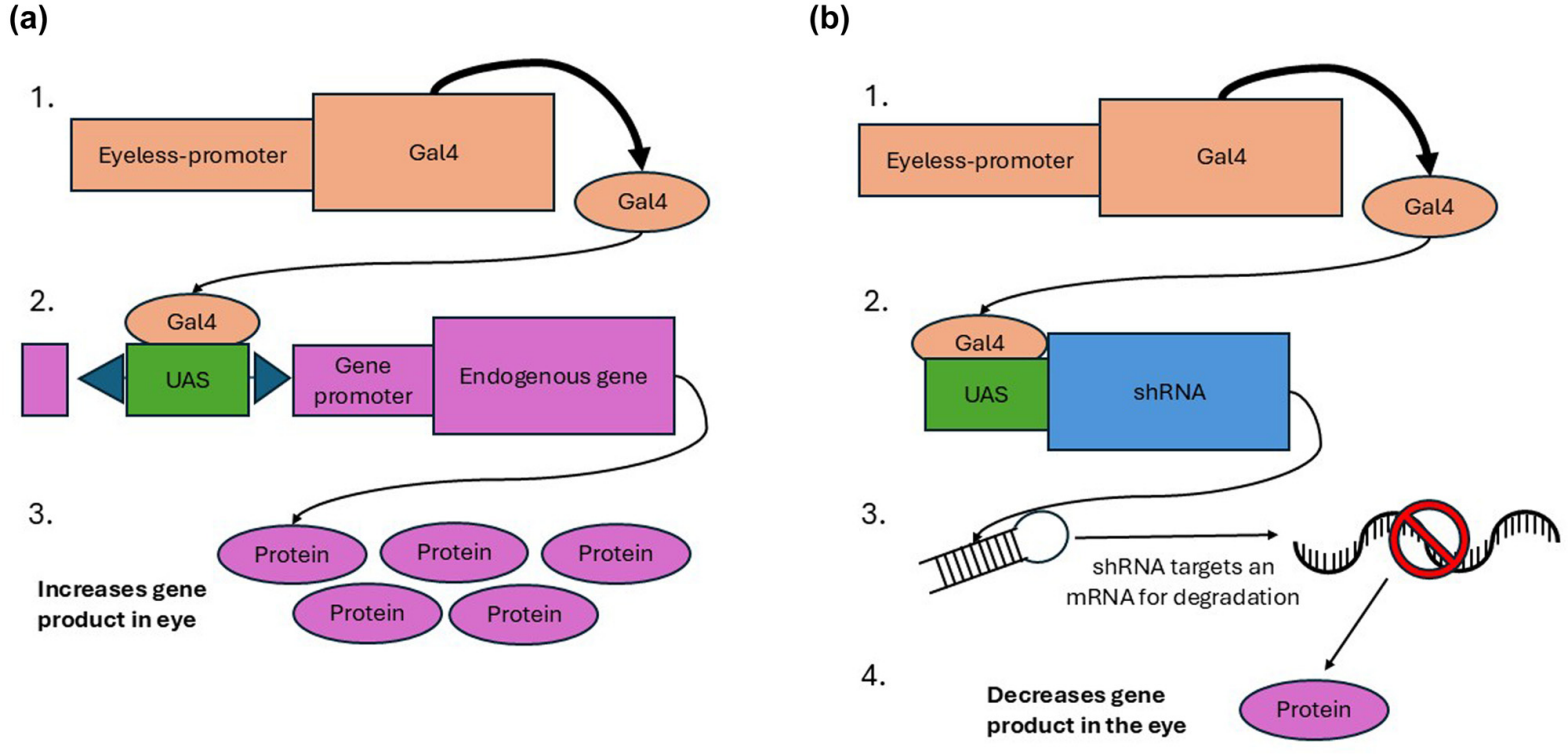
Schematic representation of overexpression and knockdown strategy in the eye. (a) Overexpression of a gene of interest in the eye. 1. (a) Gal4 encoding gene with an eyeless-promoter will express the Gal4 transcription factor in the eye (The Driver). 2. The UAS is integrated into the endogenous gene promotor via a transposable element that is stably integrated into the DNA. The Gal4 transcription factor increases the expression of the endogenous gene of interest (The Responder). 3. The overexpression of the endogenous gene of interest increases the gene product. (b) Knockdown of a gene of interest in the eye. 1. (a) Gal4 encoding gene with an eyeless promoter will express the Gal4 transcription factor in the eye (The Driver). 2. The UAS is linked to a shRNA with a complementary sequence to the gene of interest that is to be silenced. 3. The shRNA targets mRNA for degradation based on complementary sequence recognition. 4. Degradation of mRNA leads to decreased protein expression.

To further understand the role of Jazf-1 in the formation of the eye, we used two responders, creating either an overexpression or knockdown of Jazf-1 in the eye. The GAL4-UAS binary expression system was used, with an eye-specific driver, eyeless-Gal4 *(ey-Gal4)*. Our first responder was a UAS element inserted into the Jazf-1 locus [32], which had previously been reported to have a phenotypic effect when coupled with an eyeless-GAL4 driver on the *L* ^*2*^ mutant phenotype [8] (Figure 2a). A second driver was engineered to reduce expression of Jazf-1. This driver expresses a shRNA that is complementary to Jazf-1 mRNA, leading to a reduction of Jazf-1 mRNA via RNAi [38] (Figure 2b). Here, we will refer to these as Jazf-1-overexpression and Jazf-1-knockdown, respectively.

**Figure 3:**
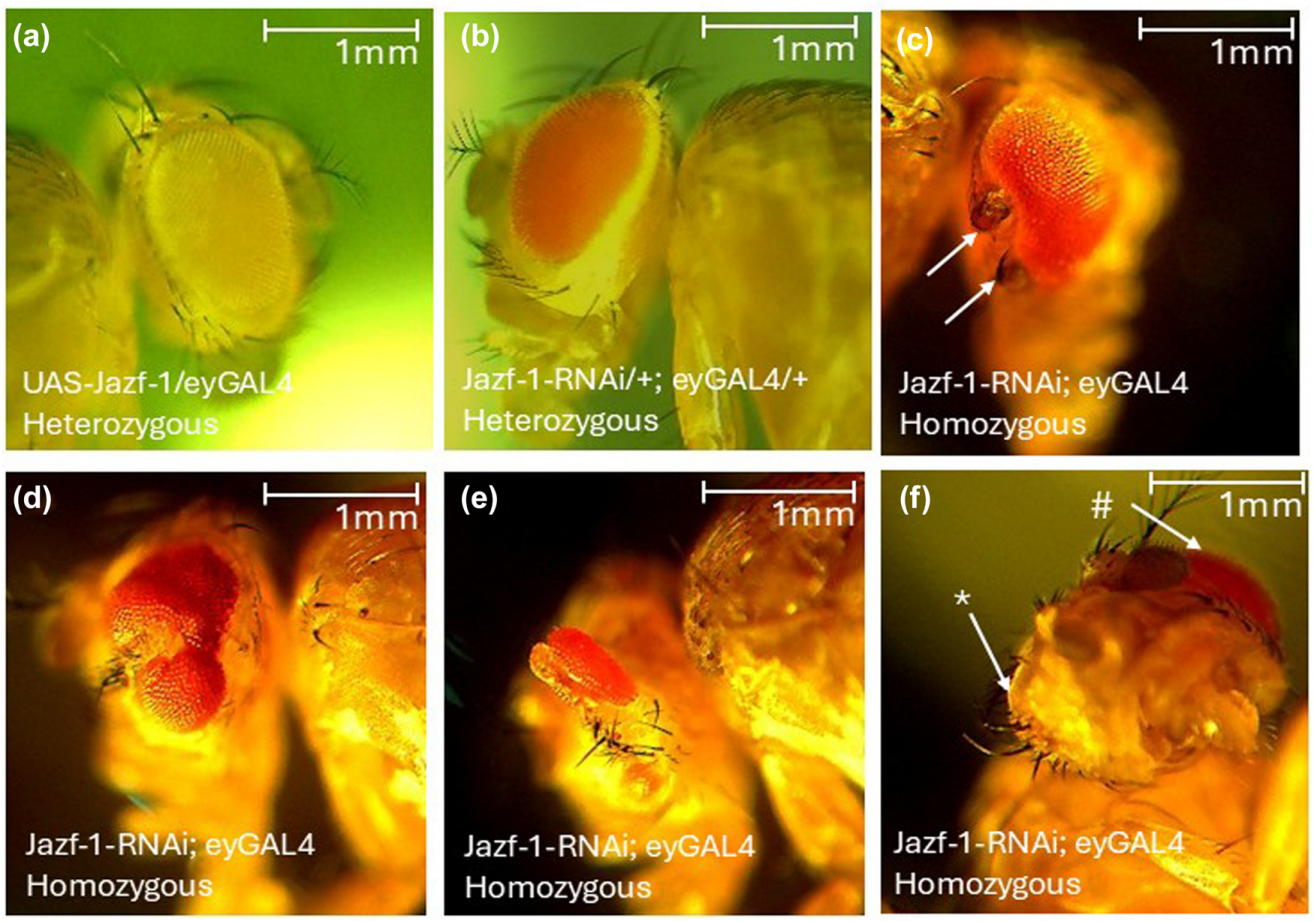
Jazf-1 overexpression and Jazf-1-RNAi driven in the eye. (a) UAS-Jazf-1 responder and eyeless-GAL4 driver. (b) Jazf-1-RNAi responder and *eyeless-*Gal4 driver as heterozygotes do not display any obvious phenotype. (c–f) Examples of Jazf-1-RNAi; eyGAL4 homozygotes display variable phenotypes. (c) Extra structures to the posterior of the eye (white arrows). (d and e) Broken sections and a smaller eye field. (f) Arrows point to two different eyes, one fully formed (#), and the other absent (*) in the same organism.

Jazf-1-overexpression and Jazf-1-knockdown in the eye lead to no appreciable change in the eye phenotype when both the driver and responder are heterozygous (Figure 3a and b). However, when both the driver and responder elements are homozygous, a phenotype is present, though not fully penetrant. Although this phenotype is not seen in all eyes, a reduction in the eyes can be observed in Jazf-1-overexpression and Jazf-1-knockdown homozygotes. Furthermore, Jazf-1-knockdown can be accompanied by irregular surfaces, eyes that are split, or extra structures. These extra structures are more commonly observed on the posterior end of the eye (Figure 3c–f). Even though heterozygous RNAi has the potential to fully eliminate Jazf-1 expression, we suspect that since Jazf-1 expression occurs early [7], and because heterozygotes would be less effective than homozygotes at quickly eliminating Jazf-1 mRNA before some protein has been synthesized, we see a more severe phenotype with homozygotes.

### 3.2 Jazf-1 reduces the *L* ^*2*^ mutant phenotype

**Figure 4:**
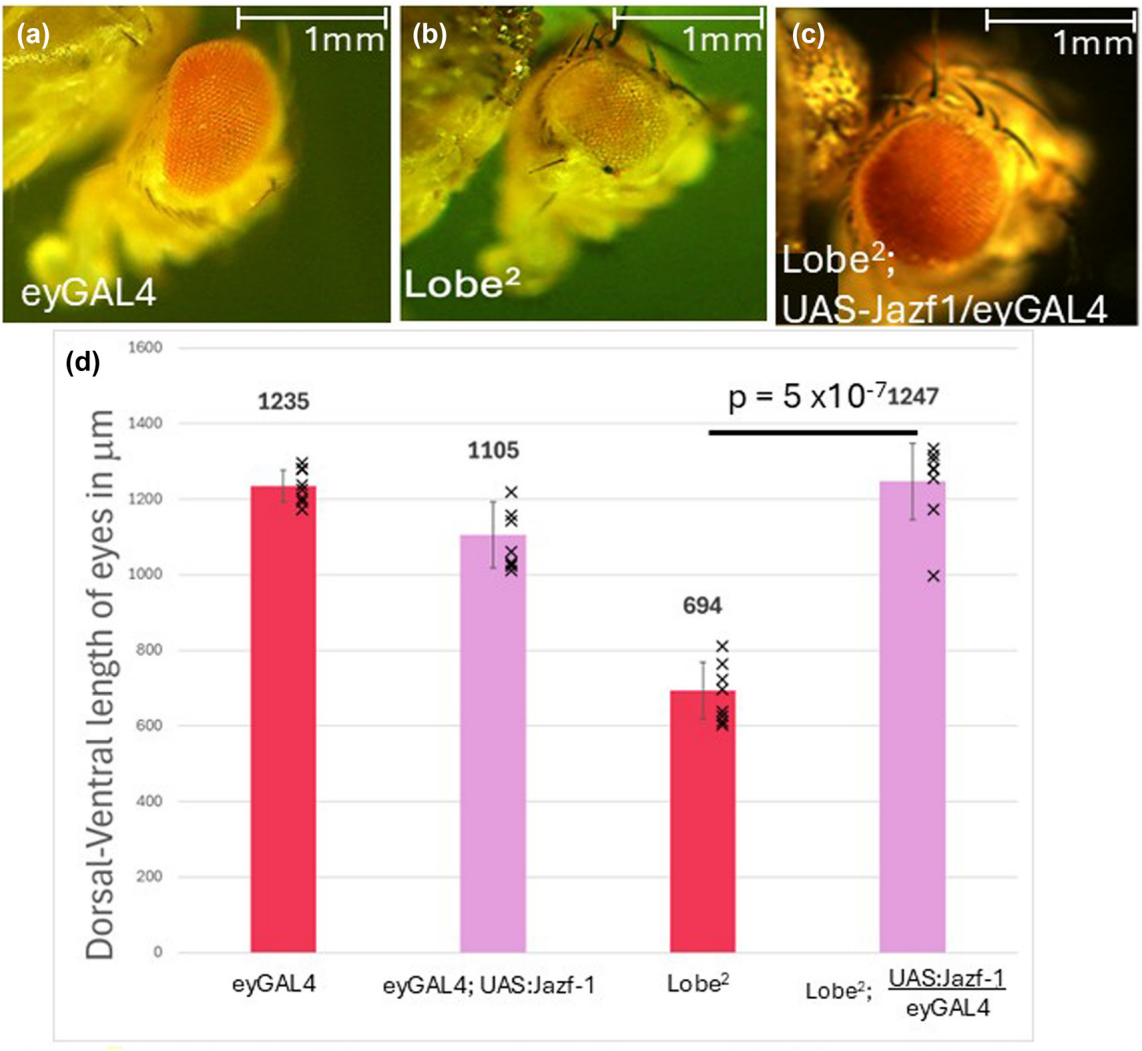
Lobe^2^ and Jazf-1 overexpression in the eye. (a–c) Representative images of *D. melanogaster* eyes. (a) Example of a line containing an eyGAL4 driver with no responder. (b) Lobe^2^ allele. (c) Lobe^2^ allele with an eyGal4 driver and UAS:Jazf-1 responder. Jazf-1 overexpression suppresses the eye phenotype of the Lobe^2^ allele. (d) Graphical representation of the eye sizes measuring the dorsal-ventral axis. All measurements are the average of 10 females; error bars are the standard deviation. A Student’s *T*-test was conducted between the Lobe^2^ and the Lobe^2^ allele rescued with a Jazf-1 overexpression in the eye (*p* = 5 × 10^−7^).

**Figure 5:**
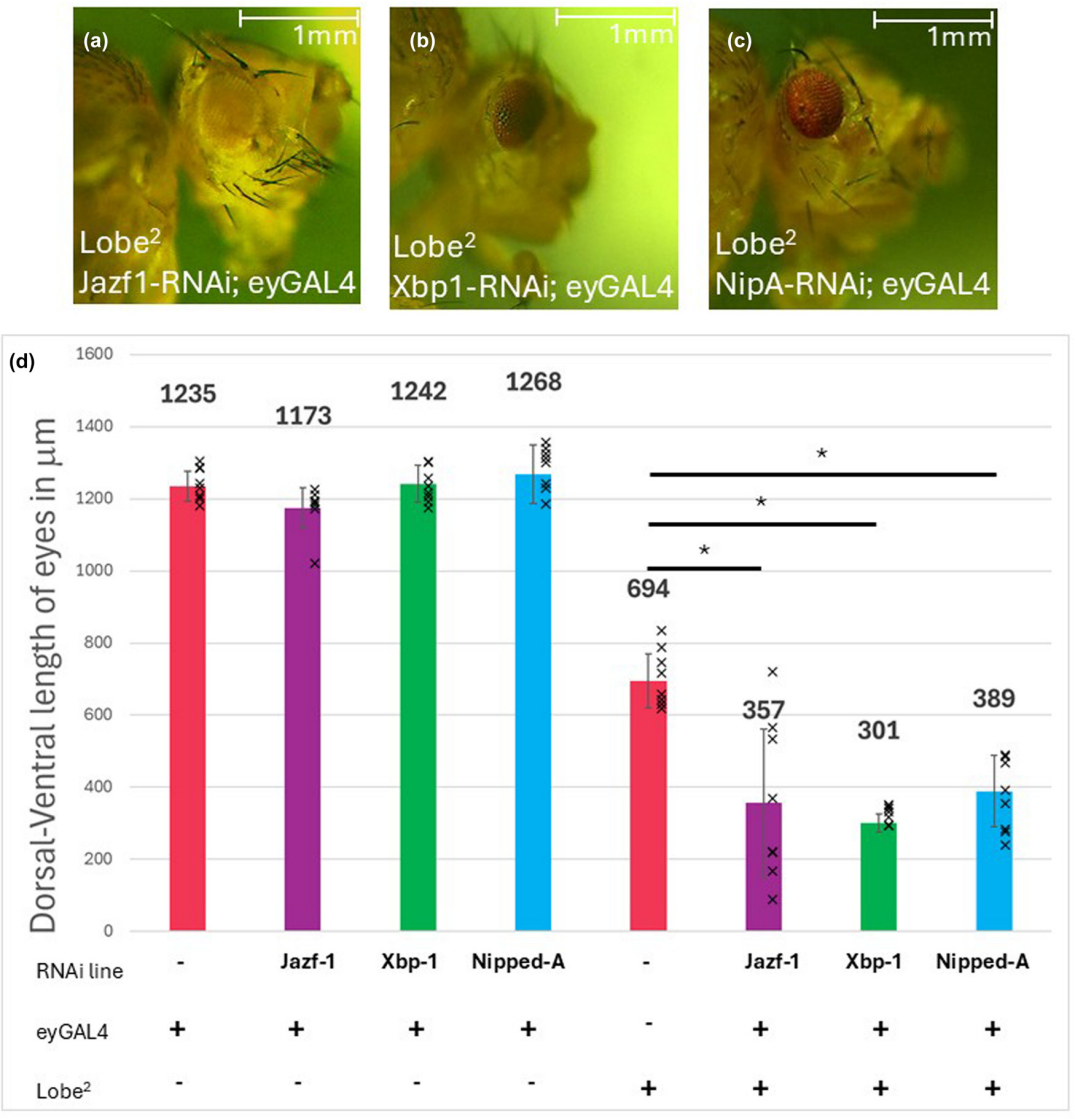
Lobe^2^ is enhanced by NuA4/TIP60 complex member knockdowns. (a–c) Representative images of *Drosophila* eyes. (a) Lobe^2^ with a knockdown of Jazf-1. (b) Lobe^2^ with Xbp-1 knockdown. (c) Lobe^2^ with Nipped-A knockdown. (d) Graphical representation of the eye sizes measuring the dorsal-ventral axis with members of the NuA4/TIP60 complexed knocked down. No appreciable difference occurred with a single driver and responder present. On a Lobe^2^ background, knockdown of NuA4/TIP60 complex members enhanced the small eye phenotype. All measurements are the average of 10 females; error bars are the standard deviation. A Student’s *T*-test was conducted between the Lobe^2^ and those with Lobe^2^ and a knocked down NuA4/TIP60 complex member showed a significant enhancement in the eye phenotype with all the members (*p < 0.0005).

It was previously reported that Jazf-1 overexpression can partially suppress an *L* ^*2*^ mutant phenotype [8]. We recapitulated that finding and demonstrated that Jazf-1 overexpression by itself does not change the eye in any appreciable manner (Figure 4). We anticipated that Jazf-1 knockdown would have the opposite effect and enhance the *L* ^*2*^ phenotype. As suspected, even a heterozygous Jazf-1-knockdown causes a significant loss of the eye when in combination with the *L* ^*2*^ mutant (Figure 5). As previously reported, Jazf-1 overexpression restores part of the normal eye phenotype on an *L* ^*2*^ mutant background [8]. Importantly, we initially observed that these phenotypes did have some degree of variation. As temperature has been previously reported to be a factor in eye development phenotypes [39], all lines that are reported were raised at 25°C, which greatly reduced the level of variation that was observed. Furthermore, females were exclusively measured to eliminate additional variation that might occur due to different sexes or gene doses from males with only one X chromosome.

### 3.3 Loss of NuA4/TIP60 histone-modifying complex proteins increase the *L* ^*2*^eye phenotype

Given the role of Jazf-1 as a member of the NuA4/TIP60 histone-modifying complex, we also tested to see if the reduction of other NuA4/TIP60 members would enhance the *L* ^*2*^ phenotype. Xbp-1 was isolated only in the *D. melanogaster* NuA4/TIP60 complex [12], and Nipped-A is a well-established core member of the complex whose human homologue TRRAP can disrupt how the NuA4/TIP60 complexes modify histones [12,15,16]. Using the shRNA UAS knockdown responders for Xbp-1 or Nipped-A with an *ey-GAL4* driver, these lines demonstrated no appreciable change in the size of the eye (Figure 5). However, siblings carrying the *L* ^*2*^ mutant background had a significant decrease in the size of their eyes when compared to the *L* ^*2*^ mutant alone (Figure 5d).

### 3.4 *Hr78* regulates the *L* ^*2*^ phenotype inversely to Jazf-1

**Figure 6:**
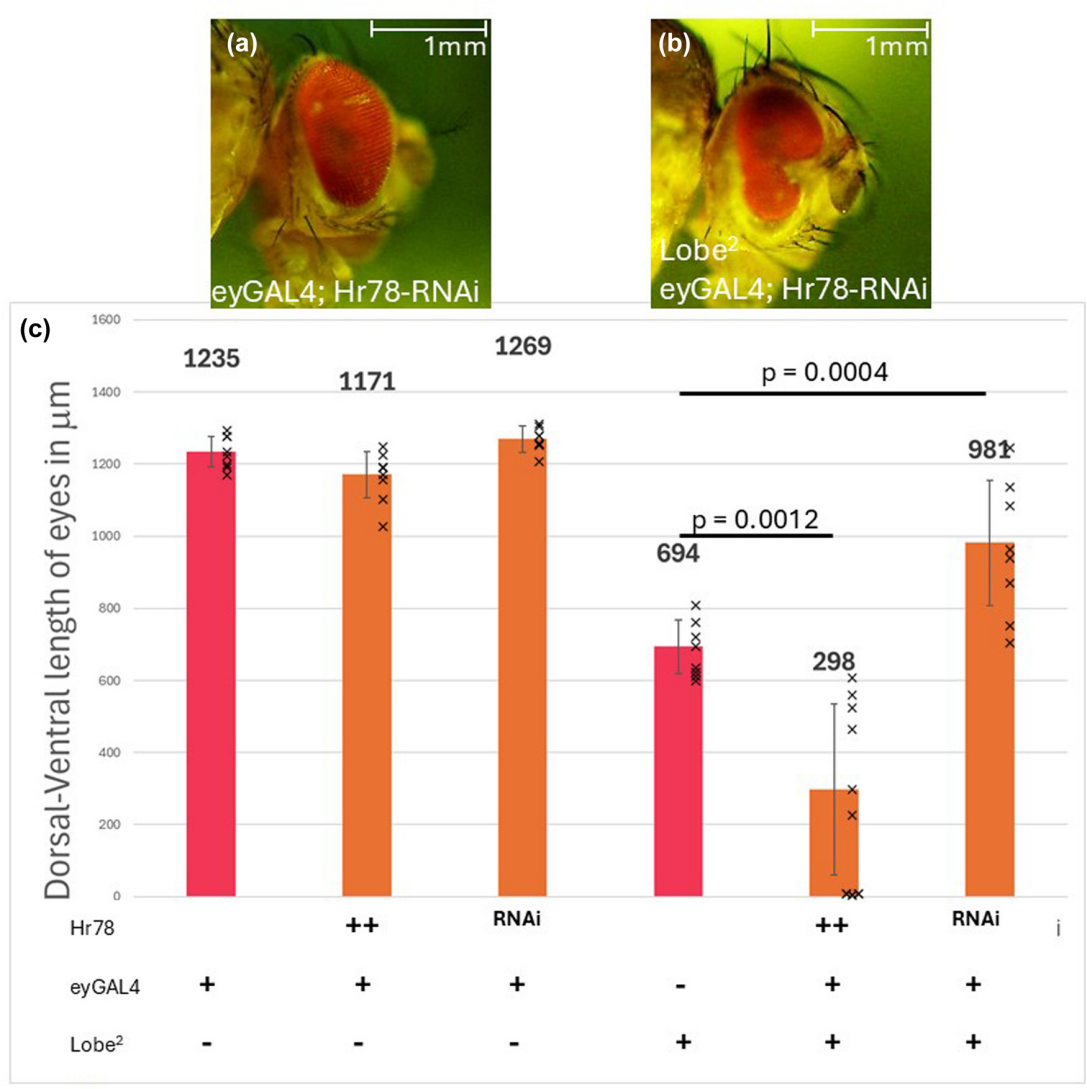
Lobe^2^ and Hr78 expression level changes influence the overall size of the eye. (a and b) Images of phenotypes resulting from Hr78 gene expression level changes with the *Lobe* ^*2*^ allele. (a) Hr78-RNAi in the eye does not significantly change the size of the eye. (b) Hr78-RNAi on a Lobe^2^ partially restores the eye. (c) Graphical representation of the eye sizes measuring the dorsal-ventral axis with variations in Hr78 expression with *Lobe* ^*2*^ alleles. All measurements are the average of 10 females, error bars are the standard deviation. A Student’s *T*-test was conducted between the Lobe^2^ and those with Lobe^2^ and variations of Hr78 expression levels.

To better understand how the NuA4/TIP60 complex might contribute to the regulation of the *L* ^*2*^ phenotype, we also investigated the nuclear receptor that Jazf-1 is known to bind, *Hr78* [13,19]. *Hr78* is already known to be expressed in the eye [31]. Again, the same GAL4-UAS binary system was used with *ey-Gal4* as the driver and either a UAS Hr78 responder or an Hr78 RNAi system responder. Surprisingly, when Hr78 is overexpressed, the *L* ^*2*^ phenotype is enhanced, and when Hr78 is knocked down by RNAi, the *L* ^*2*^ phenotype is partially suppressed. Therefore, the complete inverse of the same experiment with Jazf-1 was observed (Figure 6). Additionally, while there was variability between completely restored and partially restored eyes, the loss of Hr78 had a significant rescuing effect on the *L* ^*2*^ mutant alone (Figure 6).

**Figure 7:**
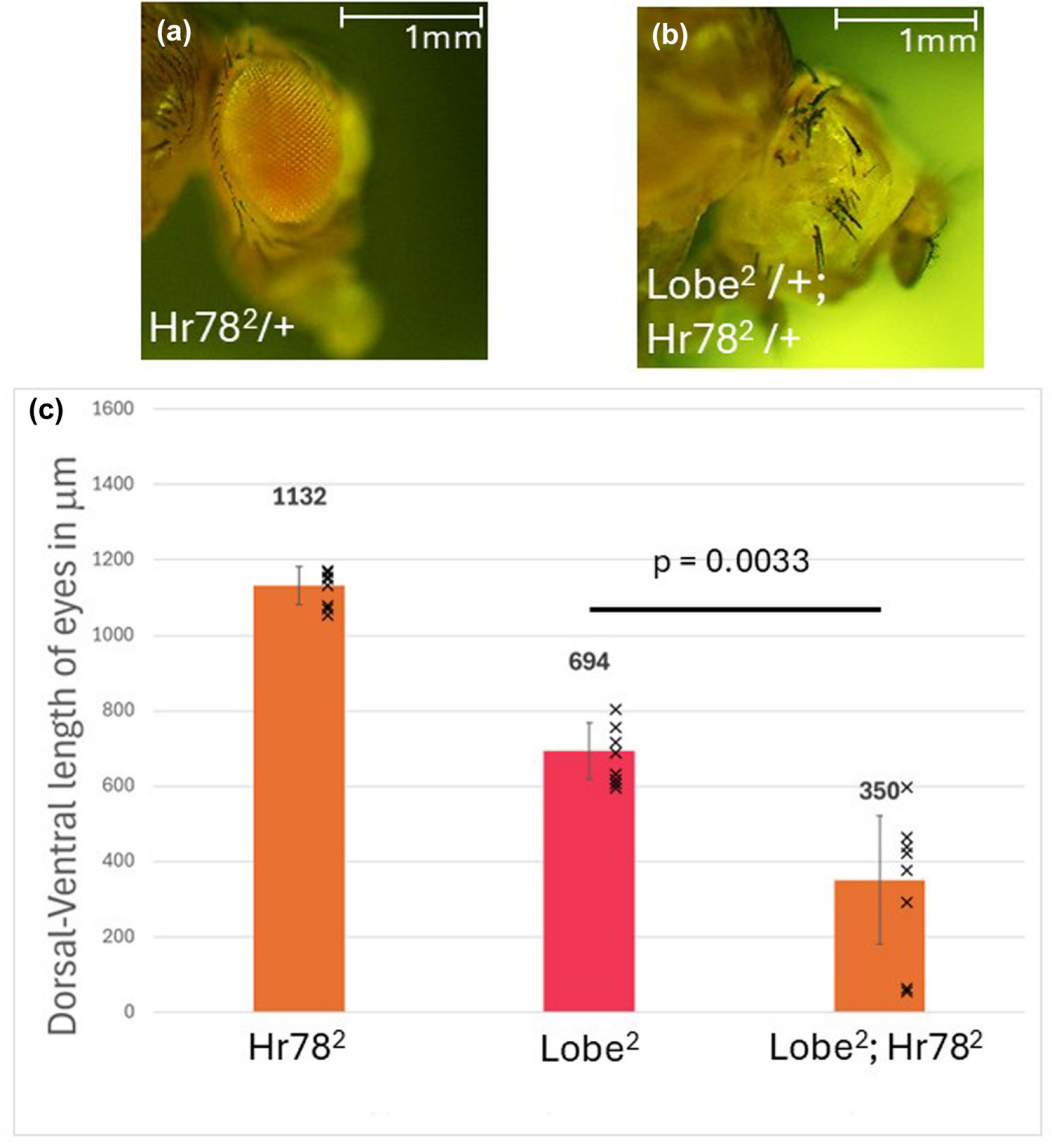
(a) Hr78^2^ heterozygous eyes. (b) *Lobe* ^*2*^ and *Hr78* ^*2*^ heterozygotes. Eyes show a decrease in the size of the eye compared with *Lobe* ^*2*^ by itself. (c) Graphical representation of the eye sizes measuring the dorsal-ventral axis. *Hr78* ^*2*^ heterozygotes display a typical eye size. When placed on an *Lobe* ^*2*^ mutant background, the size decreases, including some with no eye formed. All measurements are the average of 10 females; error bars are the standard deviation. A Student’s *T*-test was conducted between the *Lobe* ^*2*^ and the *Lobe* ^*2*^; *Hr78* ^*2*^ mutants displayed a significant enhancement in the eye phenotype (*p* = 0.0033).

To further understand the role of Hr78, we utilized an allele with an early termination stop codon, *Hr78* ^*2*^, which produces Hr78 protein with an AF-1 and DNA-binding domain (DBD) but not the AF-2 and ligand-binding domain (LBD) [31]. The AF-2 and LBD of NR2C2 have been well established as the location responsible for binding Jazf-1 [19,20,40]. Therefore, we expected that this would uncouple Jazf-1 from the *Hr78* ^*2*^ allele protein product. Unfortunately, *Hr78* ^*2*^ is homozygous lethal, which prevents the manipulation of its expression level. *Hr78* ^*2*^ heterozygotes had normal eyes, but when *L* ^*2*^ and *Hr78* ^*2*^ heterozygotes were in combination with each other, there was a significant decrease in the size of the eye compared to *L* ^*2*^ allele alone (Figure 7).

## 4 Discussion

In this study, we demonstrated that the activity of Jazf-1 and NuA4/TIP60 complex members can be measured with eye phenotypes. While using RNAi knockdowns of these genes is often insufficient to create a phenotype with a single eyGAL4 driver, in combination with a sensitized *L* ^*2*^ background, a significant reduction in eye size is observed. This provides us with an *in vivo* system to test chromatin-modifying proteins such as those in the NuA4/TIP60 complex. Second, the opposing results of NuA4/TIP60 complex members and Hr78 indicate that we can test both repressing and activating factors of gene expression using the *L* ^*2*^ background. Finally, the genetic evidence that NuA4/TIP60 complex members and Hr78 are acting in an inverse regulatory role suggests that Jazf-1 (NuA4/TIP60) is acting as a repressor through Hr78 in this context. In mammalian systems, the relationship between Jazf-1-NR2C2 as a repressor or co-activator has been in question. It is possible that these conflicting studies may depend on the tissue location and context of the gene of interest [6,17].

The expression of Jazf-1 is robust in the adult eye and the ocelli and appears to be important for proper eye formation. While a single copy of the Jazf-1 shRNA was insufficient to cause a visible change, having multiple copies and drivers resulted in a significant phenotype. Although additional copies of GAL4 drivers can also sometimes result in malformations of a tissue, these are not caused by the same eye promoter and happened at higher temperatures than what we tested [41]. It is likely that this is due to incomplete efficiency of the RNAi knockdown and that this is boosted when multiple copies of the eyGAL4 drivers and the Jazf-1 shRNA responders are present. The eyGAL4 driver and Jazf-1 shRNA responder have been verified previously, but the level of efficiency of both in combination has not yet been determined [8,42,43,44,45]. Beyond this, we are confident that the Jazf-1 responder can function as a heterozygote since Jazf-1 can modulate the *L* ^*2*^ mutant phenotype with heterozygous drivers. The data also demonstrate that the shRNA-responding elements alone were insufficient to modify the eye without the proper responder elements. While we and others [8] have found that overexpression of Jazf-1 can rescue the smaller eye, loss of Jazf-1 quickly demonstrates the sensitivity the *L* ^*2*^ background creates that is not present in an otherwise wild-type eye. Furthermore, we confirm that other members of the NuA4/TIP60 complex are required to prevent the additional shrinking of the eye. This yields genetic support to the already well-established biochemical evidence that Jazf-1 is a core component of the NuA4/TIP60 complex, and an *in vivo* context for how histone modifications can result in phenotypic changes [6,12,17,21].

A clear inverse relationship in the expression of the NuA4/TIP60 histone-modifying complex members and the nuclear receptor Hr78 is seen on the *L* ^*2*^ genetic background. A decrease in the NuA4/TIP60 complex on an *L* ^*2*^background resulted in a decrease in the overall size of the eye. To date, the only member of the NuA4/TIP60 complex that can partially rescue the *L* ^*2*^ eye phenotype when overexpressed is Jazf-1 [8]. We believe that this is because Jazf-1 provides physical contact with Hr78 that is required to suppress *L* ^*2*^, likely in conjunction with members of the NuA4/TIP60 complex. In support of this model, the Lobe phenotype is enhanced when Hr78 is increased or *Hr78* ^*2*^, an allele lacking the AF-2 domain, is used.

We believe that this unique genetic circumstance occurs because the *L* ^*2*^ allele provides an environment in the eye. The *L* ^*2*^ allele has been defined as a hypermorph, expressing *Lobe* mRNA in a tissue where it is not normally expressed [10]. Since Lobe is a transcription factor, it is likely causing overexpression of multiple genes in the *L* ^*2*^ background. We suspect that the NuA4/TIP60 complex is creating some semblance of typical eye gene expression stability by making the chromatin more typical of the normal eye. When the NuA4/TIP60 complex is compromised, the extra protection is lost, resulting in a smaller eye structure. Conversely, the addition of factors like Jazf-1, or the depletion of Hr78, seems to counteract this abnormal situation and restores the eye. We believe that this is due to the unique ability of Jazf-1 to modulate the activity of the NuA4/TIP60 complex when its expression levels are changed. Similar effects have been seen in other systems where increased Jazf-1 expression can result in changes to normal gene expression [21]. We also believe that Hr78 provides a dual role that may involve the NuA4/TIP60 complex but also other histone-modifying complexes, as well.

**Figure 8:**
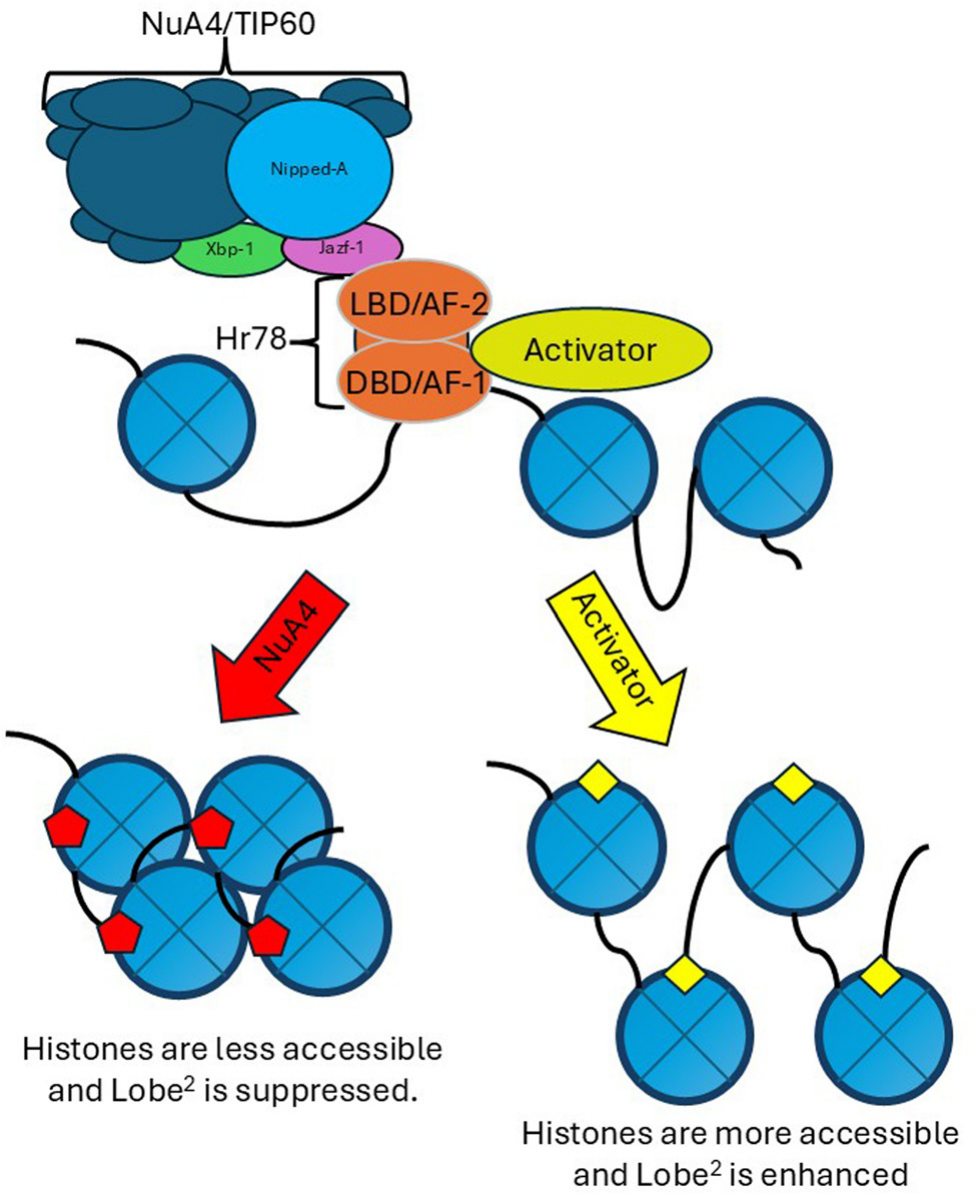
Proposed model of regulation. Hr78 acts as an anchor for chromatin modifiers. The NuA4/TIP60 complex can bind through Jazf-1 to Hr78 via the AF-2 domain. Activators can bind to Hr78, tentatively including nej/CBP through the AF-1 domain. Contact with the NuA4/TIP60 complex allows for histone modifications that typically repress expression. While the activators will enhance gene expression. The Lobe^2^ allele allows Lobe protein to be expressed in the eye and usually shrinks the eye. Changes in the chromatin modification will enhance or suppress this eye phenotype because they modify the expression changes associated with the Lobe^2^ allele.

While we can envision several different models, we propose the following model. In this, we propose that the NuA4/TIP60 complex, through its contact with Jazf-1, binds the AF-2 domain of Hr78, repressing the activity of *Lobe.* If only the AF-1 and DBD of Hr78 are present, an activator can enhance the activity of *Lobe* (Figure 8). If this is the case, we would further assume that other chromatin modifications could enhance *Lobe* activity and destabilize the eye. Simply losing a repressor without having an activator does not seem to be enough to cause this change in the eye phenotype of *Hr78* ^*2*^ heterozygous mutants on the *L* ^*2*^ background. We suspect that this activator is likely *nejire (nej)*, the human homologue to p300/CREB-binding protein (CBP). CBP has previously been demonstrated to bind to the AF-1 domain of NR2C2 and acts as an activator of gene expression [24,25]. Furthermore, an investigation into the 33 genes that were uncovered by previous screens of *L* ^*2*^ revealed that the *nej* was found to be one of seven enhancers of *L* ^*2*^ when overexpressed [8]. Since Jazf-1 and CBP modify the *L* ^*2*^ phenotype in an inverse fashion, and Hr78 can be an enhancer or suppressor of *L* ^*2*^depending on how it is manipulated, we propose that chromatin modifications are essential to the activity of *Lobe*. Whether this is at the *Lobe* locus or some downstream event that *Lobe* is affecting remains unknown. At this point, these remain untested and will need more extensive genetic analysis of multiple alleles together on the *L* ^*2*^ background. This model also generates other questions about transcriptional control mechanisms involving *Lobe*. Is it a single gene or multiple genes that lead to the *Lobe* phenotype? If a downstream gene is repressed, can it overcome the transcriptional control of *Lobe*? What other chromatin modifications can manipulate the activity of *Lobe?*

With this in mind, we envision the *L* ^*2*^ *in vivo* system has potential for the study of the effects of histone-modifying complexes on gene expression through measuring changes in the size of the eye. This could provide answers to difficult questions of how Jazf-1 and CBP help to control gene expression. Furthermore, studies have found that understanding the interplay between different histone-modifying complexes, nuclear receptors, and even signaling pathways all contribute to the understanding of diseases, such as diabetes and endometrial stromal sarcomas [21]. Using an *L* ^*2*^ background and a combination of genetic manipulations, a better understanding of the target genes could provide important key information related to the functioning of these genetic factors, thereby contributing to proper gene expression in an *in vivo* system.
